# Why are ravens smart? Exploring the social intelligence hypothesis

**DOI:** 10.1007/s10336-023-02111-6

**Published:** 2023-10-04

**Authors:** Thomas Bugnyar

**Affiliations:** 1 Department of Behavioral and Cognitive Biology, University of Vienna, Djerassiplatz 1, 1030 Vienna, Austria; 2 Konrad Lorenz Forschungsstelle, Core Faculty for Behavior and Cognition, University of Vienna, Fischerau 13, 4645 Grünau im Almtal, Austria

**Keywords:** Northern Raven, Intelligence, Social foraging

## Abstract

Ravens and other corvids are renowned for their ‘intelligence’. For long, this reputation has been based primarily on anecdotes but in the last decades experimental evidence for impressive cognitive skills has accumulated within and across species. While we begin to understand the building blocks of corvid cognition, the question remains why these birds have evolved such skills. Focusing on Northern Ravens *Corvus corax*, I here try to tackle this question by relating current hypotheses on brain evolution to recent empirical data on challenges faced in the birds’ daily life. Results show that foraging ravens meet several assumptions for applying social intelligence: (1) they meet repeatedly at foraging sites, albeit individuals have different site preferences and vary in grouping dynamics; (1) foraging groups are structured by dominance rank hierarchies and social bonds; (3) individual ravens memorize former group members and their relationship valence over years, deduce third-party relationships and use their social knowledge in daily life by supporting others in conflicts and intervening in others’ affiliations. Hence, ravens’ socio-cognitive skills may be strongly shaped by the ‘complex’ social environment experienced as non-breeders.

## Abstract (de)

Raben und ihre Verwandtschaft gelten gemeinhin als „kluge“ Vögel. Lange beruhte ihr Ruf weitgehend auf Anekdoten, erst in den letzten Jahrzehnten wurden ihre beeindruckenden kognitiven Fähigkeiten auch in Verhaltensexperimenten nachgewiesen. Während wir immer besser die kognitiven Bausteine von „Raben-Intelligenz“ verstehen, bleibt die Frage offen, warum diese Vögel derartig leistungsstarke Gehirne entwickelt haben. In diesem Artikel gehe ich dieser Frage an Kolkraben *Corvus corax* nach, indem ich gängige Hypothesen zur Evolution von Intelligenz mit rezenten empirischen Daten über Anforderungen im täglichen Leben von Raben in Beziehung setze. Die Ergebnisse zeigen, dass Gruppen nahrungssuchender Raben sämtlichen Annahmen der Sozialen Intelligenz Hypothese entsprechen: Individuen treffen einander widerholt an Nahrungsplätzen, wobei sie unterschiedliche Präferenzen für bestimmte Plätze zeigen und somit in der Gruppendynamik variieren; die Gruppen zeichnen sich durch Dominanzstrukturen und soziale Beziehungen aus; einzelne Raben erinnern sich an die Wertigkeit ihrer Beziehungen über Jahre, erkennen Beziehungen anderer und nützen ihr soziales Wissen im täglichen Leben, indem sie in Konflikte anderer eingreifen und sich in die Beziehungen anderer einmischen. Die sozio-kognitiven Fähigkeiten von Raben könnten daher stark durch die „komplexe“ soziale Umwelt geformt werden, die sie als Nichtbrüter erleben.

## Introduction

Konrad Lorenz was fascinated by the behavioral repertoire and cognitive capacities of corvids. In his first scientific papers (Lorenz 1927, 1931), he describes the richness and flexible use of social behaviors in tame, free-flying Jackdaws *Corvus monedula*, Magpies *Pica pica* and Common Ravens *Corvus corax*. One of his tame ravens also features prominently in his first popular book ‘King Solomon’s ring’ (translated from Lorenz 1949): Lorenz describes how this bird uses his name ‘Roah’ like a contact call for Lorenz, presumably to lure Lorenz away from danger. Throughout his papers and books, Lorenz kept on referring to ravens as an example of cognitive sophistication in birds.

Almost half a century later, Bernd Heinrich (1995) searched the scientific literature for what is known about raven cognition. In support of Lorenz’ view, he found > 1000 entries describing ravens as ‘smart’ birds. Yet, all but one of these reports were of anecdotal character, leaving much room for speculation about the underlying cognitive skills. In the last three decades, the picture has changed drastically: with elegant experimental designs researchers have unraveled sophisticated cognitive skills like inferential reasoning, perspective taking and future planning in ravens (Schloegl et al. 2009; Bugnyar et al. 2016; Kabadayi and Osvath 2017) and other corvids (e.g., Western Scrub Jays: Emery et al. 2007; Raby et al. 2007; New Caledonian Crows: Taylor et al. 2012; Boeckle et al. 2020). Hence, we begin to understand the cognitive building blocks that constitute corvid ‘intelligence’. What is still unclear, though, is why corvids have evolved such capacities. The aim of this paper is to take a first step towards answering the question of why ravens are smart by relating current hypotheses on brain evolution of recent empirical data on challenges faced in ravens’ daily life.

Out of several hypotheses concerning brain evolution, those related to foraging and those related to complexity of social life are particularly prominent. While the former emphasizes food distribution and/or accessibility as key factors (patchily distributed food, Milton 1981; extractive foraging, Parker and Gibson 1977), the latter considers dealing with conspecifics and maneuvering in a social network as key factors for driving cognitive evolution (social intelligence, Jolly 1966; Humphrey 1976). Note that in respect of social cognition, the focus can be on different aspects of social life like competition (Machiavellian Intelligence: Whiten and Byrne 1988), cooperation (Vygotskian Intelligence: Moll and Tomasello 2007), or information transmission (Cultural Intelligence: van Schaik and Burkart 2011). However, the common determinant of the variants of social intelligence is that predicting others’ behavior and intentions becomes increasingly difficult with variable social constellations (Whiten 2018). This leads to the assumption that the more complex social life becomes, the more individuals should invest in cognitive abilities that allow them to keep track of, and cope with, others. The problem with this intuitive assumption is that social structures in the animal kingdom are highly diverse and reflect different types of complexity (e.g., Freeberg et al. 2012; Rubenstein and Abbot 2017; Kappeler et al. 2019), which likely goes together with varying degrees of cognition. For instance, the caste-based hierarchies in eusocial species may impose different challenges and cognitive solutions than the hierarchies found in groups structured by social relationships. Indeed, it has been proposed that the essential conditions for social intelligence to emerge are those structured groups with individual-based recognition and the formation and maintenance of different types of relationships (e.g., Bergman and Beehner 2015; Kappeler 2019). In such groups, social complexity may entail (1) how many individuals interact on a regular basis (group size), (2) with whom individuals interact preferentially (social bonds), and (3) how often individuals meet and/or split up into temporary sub-groups (fission–fusion dynamics). While these measures of complexity typically refer to in-group members, the importance of out-group members or neighbors should not be underestimated and recently has received increased attention (Ashton et al. 2020).

Following this logic, I argue that to understand why ravens are smart, we need to understand their social life. However, at first glance, the social life of ravens is anything but complex: we can distinguish between two social classes, breeders and non-breeders. While breeders constitute male–female pairs that stay together for years and defend a territory for raising their offspring, non-breeders are mainly immature birds that are not restricted to a given location and tend to form loose groups at food and night roosts (Ratcliffe 1997). A similar picture can be found in many other corvids (Glutz von Boltzheim 1993). Hence, it has been argued that the social cognition of corvids is driven by challenges associated with long-term monogamous partnership rather than with conspecifics per se (relationship intelligence, Emery et al. 2007). Such an argument can be put forward not only for corvids but for monogamous species in general (Scheiber et al. 2008) and is supported by measures of relative brain sizes (Dunbar and Shultz 2007); it has received limited empirical testing on the behavioral side, though. While I acknowledge the idea of pair partners being key in understanding corvid cognition, possibly in contrast to neighbors/out-group members (compare Ashton et al. 2020), I further argue that the non-breeder state represents an additional source of social complexity. Indeed, early reports indicate some form of social structure in raven foraging groups (Coombes 1948; Huber 1991) and more recent studies described sub-groups composed of individuals with different foraging strategies (Dall and Wright 2009). Furthermore, group formation during foraging is not only a passive process, with individuals aggregating at resources of interest, but actively initiated via calls (Heinrich 1988). Note that ravens feed on highly unpredictable food sources like carcasses or kills and often face difficulties in accessing them due to competition with conspecifics and/or food defense by predators (Heinrich and Marzluff 1991, 1995). Teaming up with others could be a solution to either of the problems.

In ravens, key aspects of social cognition hypotheses (competition, cooperation, information transmission) are thus intertwined with key aspects of foraging-related hypotheses (ephemeral occurrence, restricted access). Specifically in non-breeders, foraging is a social endeavor: as a team, they may become a challenge for breeding pairs (Marzluff and Heinrich 1991) and potential predators (Vucetich et al. 2004). However, raven foraging groups are anything but stable, with individuals coming and going (Heinrich 1989). While an ‘open-group’ character has long been taken as an argument against advanced social cognition, recent theories consider high degrees of fission–fusion dynamics as cognitively challenging (Aureli et al. 2008), with the premise that group members form and maintain social relationships. Hence, for applying ideas of social intelligence to ravens, we need to examine (1) whether individuals meet repeatedly (at same or different locations), (2) whether these groups are indeed structured by different relationships, and (3) whether birds build up any form of social knowledge. I and my research group have been working on these questions over the past decades, using a mix of behavioral and bioacoustical methods. Our prime focus has been on observational studies on wild ravens in the Northern Austrian Alps. These studies are complemented with behavioral and playback experiments under field and captive conditions. Our studies are based on the following assumptions: if ravens meet repeatedly at foraging sites, they may learn about others’ attributes, which fosters individual recognition and the formation of dyadic relationships. This way, raven groups get a structured character, despite individuals having a high degree of freedom in joining/leaving (sub-)groups at a particular site. Once a structure based on social relationships is formed, several features of social intelligence may emerge—as described for mammals like primates (e.g., Cheney and Seyfarth 1990, 2007), social carnivores (e.g., Holekamp et al. 2007) or cetaceans (e.g., Connor 2007; Whitehead 2008).

### Foraging patterns and group formation

To understand how often ravens meet under field conditions, we apply two complementary approaches. First, we use a sighting/re-sighting method of individually marked ravens at a given location: the area of the Cumberland Wildpark, Grünau im Almtal, where ravens regularly snatch food from zoo animals (Drack and Kotrschal 1995). Since the mid Nineties, ravens have been habituated to the presence of human observers at the main feeding spots, i.e., the enclosures of wild boars *Sus scrofa*, bears *Ursos arctos* and wolves *Canis lupus*. Since 2008, we have been monitoring their presence at these sites on an almost daily basis following a standardized protocol.

**Fig. 1:**
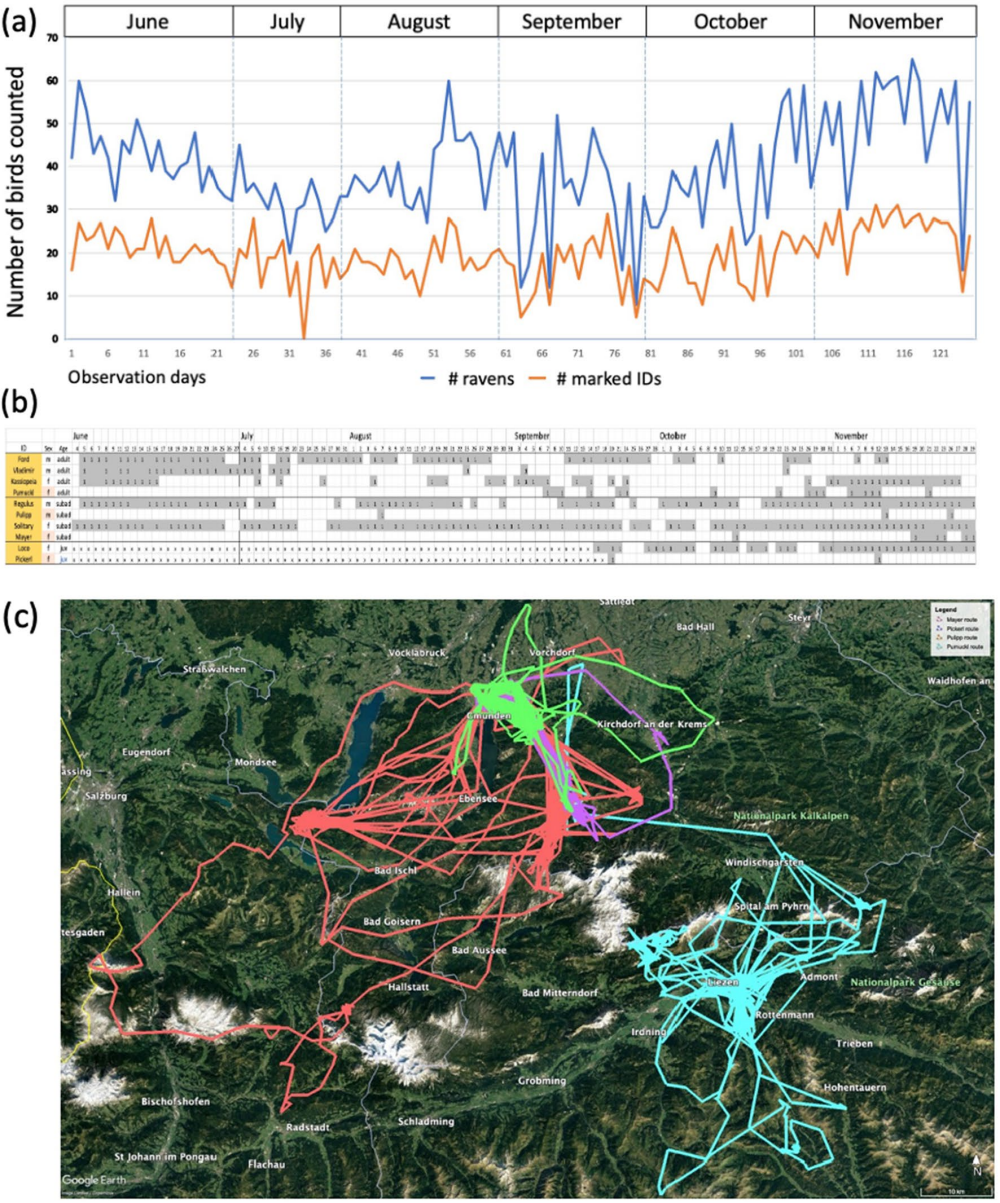
Presence patterns of Common Ravens. **a** Mean number of individuals (blue line) and mean number of marked individuals (red line) counted during feedings per day at our study site; data are plotted across 6 months (June–November 2018). **b** Presence counts of 10 marked ravens (4 adults: 2 males, 2 females; 4 subadults: 2 males, 2 females; 2 juvenile females) during the same time period; gray bars illustrate their presence at our study site. **c** GPS fixes of four of the above birds with low presence at our study site (adult female: green line, subadult female: red line; subadult male: blue line; juvenile female: pink line) across 3 months (September–November 2018)

Summarizing the findings from the presence monitoring (Braun et al. 2012), we can say the following: first, the size of raven foraging groups in the park is variable between days and across seasons (Fig. 1a). Abrupt changes in numbers for a few days (e.g., from 60 to 20 ravens to 60 ravens) point towards an opportunistic use of alternative food sources, such as carcasses or kills, when available (e.g., during hunting season in fall). Seasonal patterns (e.g., 10 + birds in summer, up to 100 birds in winter) may reflect changes in food distribution and/or accessibility across the year, e.g., because of the closing/opening of touristic areas or the pressure of territorial breeders in spring. Second, the composition of foraging groups in the park is relatively constant between days within a week, but changes across weeks with some individuals leaving and others joining (Fig. 1b). This pattern fits well to the notion of ‘open’ groups with moderate to high dynamics described from other studies in Europe and the USA (e.g., Heinrich 1989; Huber 1991; Boarman et al. 2006). As in other studies, we also find a fairly even sex ratio in the groups and an age distribution skewed towards younger birds. However, we consistently see all age-classes represented (Braun et al. 2012; Boucherie et al. 2022). Hence, foraging groups are made up not only by immature birds (juveniles in their first year: 10–20%; subadults in their second and third year: 40–60%) but also adults (3 + years; 10–30%). On an individual level, we have collected presence data from about 650 birds. Around two-third of them have been tagged as young, so that we have a fair estimation of their age due to the coloration of the inner beak (Heinrich and Marzluff 1992). Birds in their first years have a high likelihood to disappear, indicating that they suffer a high mortality risk. On average, we observe 35% of the yearly offspring till adulthood. Note that young adults typically remain in the foraging groups until they are 5–8 years. Some adults even stay non-breeders their entire life (> 10 years); others come back when they have lost their partner and/or territory. These long periods spent as non-breeders differ from those reported from other studies (review in Glutz von Boltzheim 1993; Webb et al. 2012). Possibly, our findings reflect the situation of a satiated population, where most territories are occupied and adults queue for suitable breeding opportunities, as has been described also for other territorial breeders (Ens et al. 1992; Penteriani and Delgado 2011). Finally, it is worth mentioning that not all ravens exploit our site on a regular basis: about one-third of them show up only from time to time and do not stay very long; another third pass by more regularly, every now and then, and stay for several weeks; the final third show a preference for using our site and can be observed (almost) every day over years (some individuals for > 10 years). According to the literature (e.g., Ratcliffe 1997; Heinrich et al. 1994; Webb et al. 2012), we would expect birds from the first two-thirds to be vagrant non-breeders, whereas the birds from the last third to be local breeders. However, we can find a proportion of 10–15% (confirmed) breeders in all three units; hence, the majority of vagrant and local ravens in our foraging groups are non-breeders.

To better understand where ravens go when they leave our study site, we implemented a second monitoring method that allows us to track tagged individuals over long distances (Fig. 1c). Since 2013, we have employed a subset of about 150 birds with solar-powered GPS loggers. Results show that ravens tagged at our site are recorded in the nearby region (Salzkammergut) but also in a range of several 100 kms, from Germany and Czech Republic to Italy and Slovenia (Loretto et al. 2016, 2017). Within that range, they tend to gather at specific sites for foraging. Note that the food at those sites is predominantly of anthropogenic origin like the feeding of animals at wild/game parks and farms or the ‘leftovers’ at garbage dumps, compost sites, skiing huts etc. (Jain et al. 2022). Our study site at the Cumberland Wildpark thus reflects a typical foraging location for ravens in Middle Europe. Unlike kills and carcasses, anthropogenic food sources are regularly ‘re-filled’ and thus highly predictable in space and time. Still, there are times of food ‘delivery’ (e.g., animal feedings, dumping of garbage) and/or better times of accessibility (e.g., when workers/tourists are leaving), which might explain why we typically observe ravens foraging there in groups rather than by themselves. Like with naturalistic food sources, group formation at anthropogenic sources is often accompanied by food-associated calls (Bugnyar et al. 2001), which confirms that ravens actively seek the company of others (Heinrich 1988).

Although most anthropogenic food sources are ‘re-filled’ on a daily basis, we see a huge variation between individuals in how often and how long ravens use them (Fig. 1b, c). Part of the variation can be explained by ecological factors like differences in food availability across seasons (Jain et al. 2022). However, a large part seems to be due to individual preferences: some ravens consistently exploit one or few sources, staying at given sites over years; others exploit a variety of sources, frequently changing between sites and thereby covering a large home range area (Loretto et al. 2016, 2017). Thus, the foraging groups of ravens are composed of individuals with different degrees of fission–fusion dynamics: birds with a low degree are ‘local’ to an area (compare the results from the presence data collected at the Cumberland Wildpark); birds with moderate to high degrees of fission–fusion dynamics tend to visit some or numerous sites, where they are exposed to other local ravens but also to other vagrants with moderate and high dynamics. From the perspective of locals, these vagrant ravens may be regular or irregular visitors who show up from time to time. From the perspective of vagrants, there are locals that they meet at a given location and fellow vagrants that they meet at various locations. In either case, one of the key assumptions of social intelligence is met: ravens meet repeatedly and experience a significant degree of variability and complexity.

### Group structure: dominance and bonds

To investigate whether raven foraging groups are structured by social relationships, we observe the social interactions of individuals during and outside feeding. Depending on the study, we apply focal sampling (5 min per bird) or behavioral sampling on an adlib basis per time unit (30 min). With either protocol, we can determine dominance relationships from agonistic interactions (like threats, forced retreats, fights and chases) and social bonds from affiliative interactions (like allo-preening, touching/holding body parts, and contact sitting). We face the constraint, though, that not all wild birds can be individually identified as only a proportion have been caught and marked. Hence, under field conditions, our sample is biased towards interactions between marked birds. We thus complement our studies with observations from captivity, where we can identify all individuals and calculate social networks among group members.

In captivity, ravens tend to have conflicts with several group members, whereas they engage in affiliative behaviors with only a subset of individuals (typically 1–3, sometimes up to 7; note that our captive groups consist largely of immatures and are limited to 8–15 birds). Hence, their agonistic networks are larger and more dense than the affiliative networks (Kulhaci et al. 2016), which fits the pattern of many avian and mammalian species forming structured groups (e.g., Croft et al. 2008). If we calculate the mean (± SD) number of interaction partners between marked birds under field conditions per year, we see a similar picture: wild ravens engage in agonistic interactions with 8–12 birds (females: 8.4 ± 2.2, range 0–49; males: 11.6 ± 3.5, range 0–70) and affiliative interactions with 1–2 birds (females: 1.4 ± 1.3, range 0–9; males: 1.6 ± 1.2, range 0–8). The small number of affiliation partners supports the notion that ravens focus on a few individuals per time, even when the number of potential partners is not restricted. Affiliative interactions can be exchanged between birds of same and different sex as well within and across age-classes (Braun et al. 2012; compare Boucherie et al. 2020 for captivity). However, affiliative relationships are typically composed of male–female dyads, whereby the identity of the affiliation partners may change between seasons and/or years (Braun et al. 2012). This finding is corroborated by observations at other sites (see Glutz von Bolzheim 1993) and, from a functional point, supports the view of non-breeders testing potential long-term partners.

In our captive groups, ravens consistently form a dominance hierarchy (Boucherie et al. 2022), which has been reported also from studies, in which ravens were temporarily kept in free-flight (Gwinner 1964) or wild ravens were temporarily restrained to an aviary (Marzluff and Heinrich 1991). Furthermore, there have been speculations that wild ravens may form dominance rank hierarchies at commonly used anthropogenic foraging sites (Huber 1991). Indeed, our recent analysis of raven foraging groups at our study site reveals a clear steep dominance rank hierarchy (Boucherie et al. 2022). Note that about 100 marked ravens were involved in each of the two data sets of this field study (2008–10; 2017–19), representing about half of the population using the park at those times. Assuming that interactions with unmarked birds follow the same pattern as with marked birds, this suggests that our ravens can deal with the dominance status of at least 200 individuals. Recall that not all of the ravens are present in the foraging groups at our study site all the time; in fact, two-thirds of them show medium to high degrees of fission–fusion dynamics and pass by only occasionally. As a whole, the evidence suggests that ravens have a good memory of individuals and their rank, which is reinforced during facultative encounters. Alternatively, they might use observable cues (like body size) or behavioral expressions (like self-aggrandizing displays (Lorenz 1940; Gwinner 1964) that are related to sex, age and/or bonding status) to judge the dominance status of others (Heinrich 1989).

**Fig. 2:**
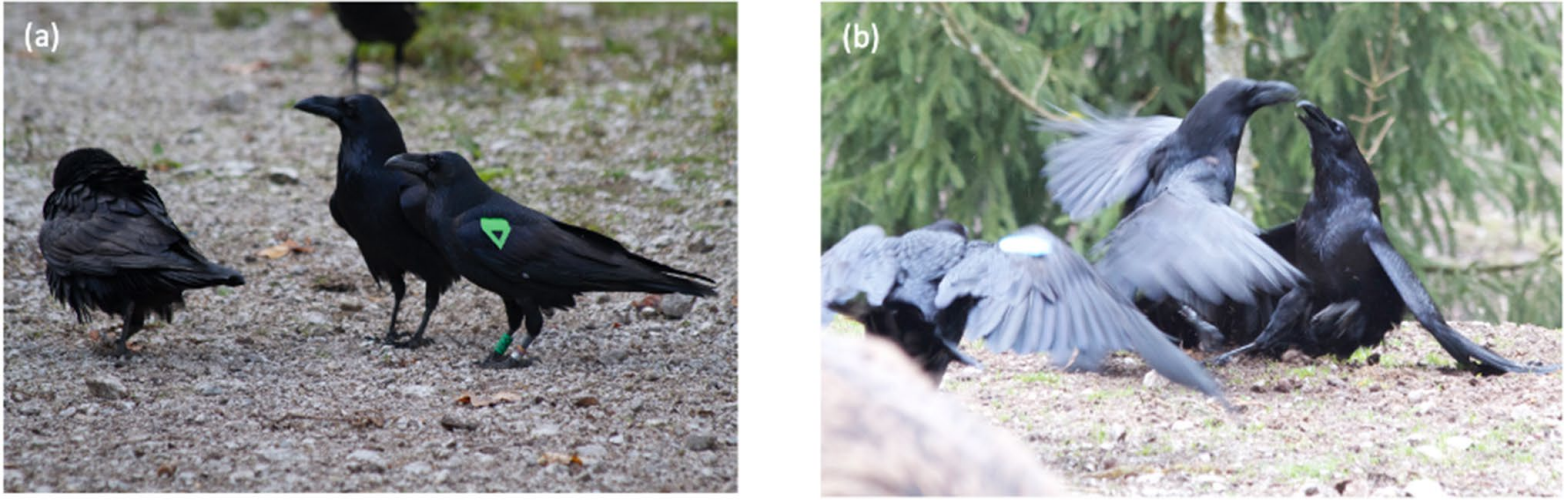
Passive (**a**) and active (**b**) social support in wild Common Rravens. **a** The approach of the bird on the right (marked with green wing tag) causes the bird on the very left to retreat. **b** The bird on the left front (marked with blue wing tag) is about to intervene in the ongoing fight, attacking either the aggressor (middle) or victim (right). Copyright: Thomas Bugnyar, Georgine Szipl

Under captive and field conditions, males tend to outrank females, older birds tend to outrank younger ones and bonded birds tend to outrank non-bonded birds (Braun et al. 2012; Boucherie et al. 2022). These effects indicate that ravens achieve their rank in the dominance hierarchy primarily due to their competitive abilities such as physical strength (males are larger) and fighting experience (older birds have an advantage) and—to some extent—also due to (repeated) social support by other ravens. Social support refers to a bystander intervening in a conflict, either by actively helping (attacking one of the combatants) or passively by its mere presence in a conflict situation (e.g., one of the combatants is backing off when the supporter approaches; Fig. 2). Providing active support has been considered fundamental to social intelligence, especially the Machiavellian version (Whiten and Byrne 1988). In ravens, active social support occurs in about 5–10% of agonistic interactions and is used selectively, depending on the individual characteristics of the birds involved in the conflict and/or the relationship of the supporter to one of the combatants. For instance, active aggressor support is typical for younger males, who use conflicts of dominants to challenge individuals higher than themselves. Victim support can result as a byproduct from dominants attacking young aggressors (likely as a response to them challenging the hierarchy) or directly from individuals that mutually support each other. Note that aggressor support is shown about twice as much as victim support, likely because the latter poses a higher risk of injury to the supporter, particularly when conflicts are severe (fights, chases). Yet, getting help as victim likely changes the outcome of the conflict (Szipl et al. 2017) and, if provided repeatedly, may lead to a rank dependent on the supporter’s help. Dependent ranks have already been described by Lorenz (1931), (1988) and appear to be common among pair partners in long-term monogamous species like many birds (e.g., Scheiber et al. 2005) and in kin-structured groups (e.g., matrilines in old-world primates, social carnivores, cetaceans; Cheney and Seyfarth 1990, 2007; Holekamp et al. 2007; Whitehead 2008). Long-term collaborations in conflicts are also referred to as alliances, which contrast with facultative short-term coalitions (de Waal and Harcourt 1992). Applying this terminology, we see both strategies in raven foraging groups: facultative coalitions and long-term alliances. Note that the latter occurs not only between breeding pairs, but also between non-breeders with high-quality relationships, i.e., social bonds.

Following the concept developed in primatology (Hinde 1976), we refer to social bonds in ravens when individuals exchange affiliative behaviors reciprocally over time. Recall that such an exchange is not restricted to adults but can be seen in birds of all age-classes (Braun et al. 2012), whereby siblings tend to be preferred partners in young ravens (Kulhaci et al. 2016). The amount and equity of providing affiliative services may differ substantially between bonding partners and likely reflects the value of the dyads’ relationship (Fraser and Bugnyar 2010a). In our wild population, for instance, females tend to provide substantially more allo-preening than their male counterparts during bond formation. At the same time, they start winning conflicts during foraging, suggesting that they immediately profit from the presence of a (potential) bonding partner. Males also profit from bonding by winning more conflicts, but not before they consistently reciprocate affiliative services to their partner (Braun et al. 2012). In captivity, bonded ravens tend to reconcile their conflicts (Fraser and Bugnyar 2011) and consistently provide bystander affiliation to each other post-conflict (Fraser and Bugnyar 2010b). These patterns fit well to the conflict management described in social mammals, particularly primates (Aureli and de Waal 2000), and indicate that ravens might be interested in repairing their relationships when damaged by conflicts, and in alleviating stress of their bonding partners caused by conflicts with others. Post-conflict behaviors have also been described for other corvids in captivity (e.g., Seed et al. 2007; Sima et al. (2018); Logan et al. 2013) but we do not have experimental evidence for the use of those strategies under field conditions yet (Lee et al. 2019). However, we know from primate studies that post-conflict bystander affiliation can serve different functions and may differ substantially between species and even within (captive) populations of the same species (Koski and Sterck 2007; Fraser et al. 2008). Still, the fact that wild and captive ravens support each other during and (possibly) after conflicts when bonded, provides a strong case that forming and maintaining relationships are of immediate value to them in daily social life. In this respect it is surprising is that not all birds have bonding partners; in fact, about half of the ravens in our foraging groups have no partners at all over the time course of a year and sometimes over years.

### Social knowledge and cognition

To investigate whether ravens recognize and memorize individuals and their social relationships, we make use of their elaborate acoustical communication via playback experiments, inspired by the seminal work of Cheney and Seyfarth (1990, 2007) on primates. We focus on a subset of calls that can be linked to a specific environmental context (like the presence of food and predators) or social context (like self-advertisement or appeasement) and which typically contains information about the sender’s identity, sex and age class (Boeckle et al. 2012, 2018). Furthermore, we apply behavioral observations to examine tactics that imply the use of social knowledge.

If we first focus on captive ravens, where we have full control over the exposure to conspecifics (whom they have or have not met, and thus can or cannot know), we have shown that they remember former group members and their relationship valence over years (Boeckle and Bugnyar 2012). Specifically, adult pair-housed ravens respond stronger to the playback of contact calls (‘rab’) of former group members (individuals they were kept with as non-breeders) as compared to unfamiliar birds matched for sex and age, and they modulate their call response to familiar birds, depending on whether they were former ‘friends’ or ‘foes’ (birds they had a close affiliative relationship with or not). These results are in line with our hypothesis that ravens build up social knowledge about group members in the non-breeder state. That they can retain this information for years fits to the moderate to high fission–fusion dynamics ravens experience under field conditions.

Our field studies corroborate that ravens discriminate between social categories, and possibly individuals, in daily life situations. For instance, foraging ravens vary strongly in calling at food (‘haa’), which can be explained by social factors like the presence/absence of territory holders (Marzluff and Heinrich 1991) but also by individual characteristics like the birds’ age, sex and vagrancy status (Boeckle et al. 2018; Szipl et al. 2015). Using a paired playback design, we have shown that ravens foraging in the Cumberland Wildpark preferably approach loudspeakers broadcasting female callers, but only if those are local birds, i.e., familiar to them (Szipl et al. 2015). On the production side, we note that adult females call at food when they are all by themselves at the foraging site (Sierro et al. 2020), suggesting that they address their bonding partners (i.e., want them to come).

A key question in respect of social knowledge is whether individuals represent not only their own relationships but also the relationships between other group members (third parties; Cheney and Seyfarth 1986; Tomasello and Call 1997). Indeed, when we play back a simulated conflict between two familiar individuals to subadult ravens in captivity, they respond stronger to playbacks in which the outcome reflects a violation of the hierarchy as compared to outcomes that are in line with the existing hierarchy (Massen et al. 2014a). Interestingly, ravens respond to such simulated rank reversals not only when those concern members of their own group but also members of the adjacently kept group. These findings clearly show that ravens can represent the rank relationship between other individuals, and they can possibly do so by mere observation.

In our playback experiment, we make use of the fact that ravens utter specific calls when they are challenged by a dominant (Gwinner 1964). These calls may primarily serve to appease the aggressor but also alert and/or attract nearby ravens (Heinrich et al. 1993). As with food calls, appeasement calls vary strongly between individuals and context. Given their functions, we can expect victims of aggression to call more when the conflicts are severe (to appease the aggressor) and/or when there are potential allies in the audience (to seek help). Indeed, we see that victims modulate their calling rate according to the audience composition: they increase calling when close kin are present but decrease calling when their aggressors’ bonding partners are present (Szipl et al. 2018). The former indicates that (young) ravens take into account their own relationships when calling for help; the latter indicates that they also take into account the relationship between others, as they seemingly try not to alert the aggressor’s partner. Hence, the context of social support seems promising to probe for third-party understanding in wild ravens. Note that ravens intervene not only in others’ conflicts but also in others’ affiliative interactions (Massen et al. 2014b), whereby they selectively target individuals that are about to form bonds (i.e., start reciprocating affiliative behaviors). Recall that bonded birds provide both active and passive support, leading to a higher probability of winning conflicts and eventually a rise in rank (Braun et al. 2012). We thus interpret the selective interventions in early stages of bonding as attempts to prevent those birds from becoming alliance partners and thus possible competitors. This would mean that ravens not only come to understand others’ relationships but also try to prevent some to form. Such tactical moves have been first reported for chimpanzees and referred to as ‘politics’ (de Waal 1982).

## Summary and outlook

Taken together, our studies reveal that (1) ravens meet repeatedly at foraging sites, either at the same location or at different locations; (2) foraging groups are composed of individuals with different site preferences and thus degrees of fission–fusion dynamics; nevertheless, the groups are structured by dominance rank hierarchies and social bonds; (3) ravens memorize former group members and their relationship valence over years, deduce third-party relationships and use their social knowledge in daily life by supporting others in conflicts and intervening in others’ affiliations. Hence, ravens meet our assumptions concerning social foraging and intelligence.

Before drawing conclusions, let me try to put each of the key results into context: given that our findings come from ravens that almost exclusively feed on food sources of anthropogenic origin, we need to be open about the possibility that the observed patterns could be a recent development with limited implications from an evolutionary perspective. Wild parks or skiing huts, for instance, have not been operating much longer than 50 years. Yet, ravens are scavengers and, as such, prone to utilize food made accessible by other species (Stahler et al. 2002; Vucetich et al. 2004), humans being no exception (Heinrich 1989). In fact, there is a long history of ravens exploiting resources provided by humans over hundreds and possibly thousands of years (Marzluff and Angel 2005; Baumann et al. 2023). Hence, ravens in Middle Europe might have simply adjusted to the type of resources offered in today’s landscape but their regular meetings at foraging sites could possibly reflect a species-general feature typical for their scavenging lifestyle. In support of this idea, a recent project in Yellowstone National Park shows that also under ‘naturalistic’ conditions (with limited impact by humans), ravens rely to a great extent on human subsidies, forming groups at anthropogenic food sources especially during winter (Ho et al. 2023). We also see similar patterns of group formation and composition in carrion and hooded crows *Corvus corone* and *C. cornix* foraging in Zoo Vienna (Uhl et al. 2019). Hence, our findings may apply generally to corvids with a similar foraging ecology and social structure than common ravens.

Raven groups at anthropogenic food sources can be interpreted as aggregations, with birds ending up using the same food source independently from each other. However, if raven groups were only aggregations at foraging sites, we would not expect them to signal their motivation to feed via specific calls, nor would we expect them to display a dominance rank hierarchy in competition for food. Yet, our ravens do use ‘haa’ calls before they start foraging at the enclosures of zoo animals, which indicates that, like at carcasses, individuals actively coordinate for approaching food (Heinrich 1988). During feeding, ravens repeatedly get into conflicts with each other, whereby they show a clear dominance rank hierarchy despite regular changes in the group composition. Forming and keeping track of dominance relationships thus works under conditions of moderate (to high) fission–fusion dynamics. From a cognitive point of view, this fits well to the fact that several corvid species tested on transitive inference tasks in the lab are capable of predicting rank relationships (Bond et al. 2003; Lazareva et al. 2004; Paz-y-Mino et al. 2004; Mikolasch et al. 2013). These results are in line with those from species as diverse as wasps *Polistes* sp. (Tibbets et al. 2019), fish *Astatotilapia burtoni* (Grosenick et al. 2007), geese *Anser anser* (Weiß et al. 2010) and primates *Macaca mulatta* (Gazes et al. 2012); together they support the conclusion that transitive inference is one of the cognitive building blocks that emerge when animals live in social groups structured by dominance ranks (MacLean et al. 2008; Fernald 2014; Doi and Nakamura 2023).

At first glance, it may be of little surprise that raven foraging groups are also structured by social bonds, given that they form long-term monogamous breeding pairs (compare Emery et al. 2007). However, raven foraging groups consist to a large extent of immature birds (that per definition do not form breeding pairs) and adults without a territory (that do not have the opportunity to breed). Yet, they form social bonds that are hardly distinguishable from pair bonds of territorial breeders, except that they appear to be less stable over time. The importance of ‘personal friendship’ was also noted by Lorenz (1931), as his tame ravens treated human interventions according to context. Under field conditions, the social bonds of (non-breeding) ravens seem to function as alliances in conflicts (compare de Waal and Harcourt 1992). Possibly, the social support comes as a by-effect of bonding, as it has been described also for other long-term monogamous species (e.g., Black and Owen 1986; Scheiber et al. 2005; Morales et al. 2022). However, ravens provide a decent amount of support also to non-bonded individuals, either by helping aggressors in beat-ups or by challenging aggressors before they could attack another bird. Such temporary coalitions speak for a tactical use of third-party interventions (Whiten and Byrne 1988), whereas the reciprocal support in alliances may be based primarily on emotions (Schino and Aureli 2021). As with most other species, the cognitive underpinnings of both types of social support are speculative and would need to be investigated experimentally. The same is true for post-conflict management, which seemingly emerge with the importance of social bonds across a variety of species (Fraser et al. 2009) and may, but does not have to, be based on sophisticated cognitive mechanisms (Cordoni et al. 2023).

Given the composition and dynamics of foraging groups, we can argue that ravens do face a ‘complex’ social life. According to the social intelligence hypothesis, we may thus expect them to build up social knowledge about group members, which is in line with the results from our playback experiments under captive and field conditions. Memorizing individuals and their relationships over years fits well to what is known from other social animals (review on individual recognition: Yorzinski 2017; social memory: e.g., McComb et al. 2000; Bruck 2013). Possibly, long-term memory for group members is the rule rather than an exception in species living in structured social groups. This said, it is often unclear if the animals’ memories are based truly on individual recognition or rather on refined class-level recognition (Tibbets and Dale 2007). For instance, the ravens in our experiment might have remembered social categories (in-group vs. out-group members; affiliates vs. non-affiliates). However, we know from a study by Kondo et al. (2012) that large-billed crows *Corvus macrorhynchos* match the visual image and acoustical call of group members, but not of unfamiliar individuals, in a cross-modal design. Hence, we have experimental evidence for individual recognition based on mental representation in a closely related corvid species. Moreover, our simulated rank reversal experiment would not have worked, if the ravens would not be capable of recognizing specific individuals and their rank relationships. Such a third-party understanding is considered as an important building block for advanced social cognition (Tomasello and Call 1997), as it allows a high flexibility in social maneuvers. However, third-party understanding also leads to a high information load and it is still debated how well it is expressed in different species (see Bergman 2010 within primates; Lee et al. 2019 within corvids). The selectivity in requests for social support by victims of aggression suggest that wild ravens are capable of tracking the affiliation status of other ravens. Together with the results from playback experiments on simulated rank reversals, we may conclude that ravens can track others’ dominance and affiliation status. The selective interventions in affiliative interactions by bonded birds indicate that ravens may even go a step further and attempt to manipulate the formation of bonds in other ravens, which may be referred to as ‘politics’ (de Waal 1982). Aside ravens and some primates (Mielke et al. 2017), interventions in affiliative behaviors have been reported also from domestic horses *Equus caballus* (Schneider and Krueger 2012), but the strategic character of those maneuvers is debated. Again, experiments would be needed to test for the cognition underlying those tactics.

In conclusion, socially foraging ravens fulfill several criteria for applying social intelligence (sensu Whiten and Byrne 1988). They do show sophisticated behaviors and cognitive skills in the social domain that are comparable to those reported from other socially complex species, notably primates. Although our findings support the idea of convergent evolution of socio-cognitive traits in distantly related taxa (Emery et al. 2004), we still need to test for the cognitive mechanisms underlying (some of) these traits in either of the taxonomic groups. As a final point, I would like to highlight the enormous variation we see among individuals in how they cope with (the same) challenging situations in everyday life. Understanding the causes and consequences of this variation (e.g., nutritional/social/developmental stress: Nowicki et al. 2002; Sachser et al. 2011; Boogert et al. 2014; social competence: Taborsky and Oliveira 2012) would be an important next step towards an integrative view of raven social cognition, much in the sense of Tinbergen (1963).
